# Glandular Trichomes Serve as the Primary Storage and Functional Sites of Oridonin in *Isodon rubescens* Leaves: Spatial Localization, Quantitative Validation, and Bioactivity Correlation

**DOI:** 10.3390/cimb48090887

**Published:** 2026-08-31

**Authors:** Jimeng Zhang, Xiaoyu Su, Yao Sun, Chunming Li, Yongliang Yu, Yaling Yang, Yiwen Cao, Lina Wang, Lei Li, Dandan Lu, Mengfan Su, Zhengwei Tan, Huizhen Liang

**Affiliations:** 1 Institute of Chinese Herbal Medicines, Henan Academy of Agricultural Sciences, Zhengzhou 450002, China; zjimeng2026@163.com (J.Z.); suxiaoyu_2014@163.com (X.S.);; 2 Provincial Key Laboratory of Conservation and Utilization of Traditional Chinese Medicine Resources, Zhengzhou 450002, China

**Keywords:** *Isodon rubescens*, glandular trichome, oridonin, desorption electrospray ionization mass spectrometry, cytotoxicity

## Abstract

As a non-volatile diterpenoid, oridonin represents the major bioactive compound in the medicinal plant *Isodon rubescens* (Hemsl.) Hara. This compound exhibits a broad range of pharmacological activities, including potent anticancer effects against various tumor types, as well as antibacterial and anti-inflammatory properties. Although the pharmacological properties of oridonin have been extensively characterized, its exact tissue-level distribution in leaves has yet to be elucidated. In this study, histochemical staining, desorption electrospray ionization mass spectrometry imaging (DESI–MSI), and dichloromethane-targeted extraction coupled with high-performance liquid chromatography (HPLC) were employed to determine the cellular distribution of oridonin in *I. rubescens* leaves. Histochemical staining with Hydrochloric acid–vanillin (HCl–vanillin) revealed intense fluorescence signals exclusively in peltate glandular trichomes, with no detectable fluorescence in mesophyll cells. DESI–MSI analysis showed that the characteristic ion signal of oridonin (*m*/*z* = 387.15) exhibited a punctate distribution pattern closely matching the spatial arrangement of glandular trichomes. Quantitative HPLC analysis demonstrated that oridonin content in glandular trichome extracts accounted for 77.44% of that in whole-leaf extracts, whereas mesophyll extracts contained only 10.20%, suggesting that glandular trichomes serve as the primary storage site. Furthermore, bioactivity assays revealed that glandular trichome-enriched extracts exhibited significant antibacterial activity against *Bacillus subtilis*, *Micrococcus luteus*, and *Staphylococcus aureus*, and showed cytotoxic effects on A549 human lung adenocarcinoma cells, with activity levels positively correlated with oridonin content. These convergent lines of evidence provide evidence that glandular trichomes are the main accumulation and storage sites of oridonin in *I. rubescens* leaves, and that trichome-stored oridonin constitutes the primary material basis for the antibacterial and cytotoxic activities of this plant. This study provides a cellular-level basis for the quality evaluation and breeding of high-oridonin *I. rubescens* varieties.

## 1. Introduction

*Isodon rubescens* (Hemsl.) Hara is a medicinal plant whose dried aerial parts are officially recorded in the Chinese Pharmacopeia, and oridonin is recognized as a key quality control marker for this herbal material. It is a perennial shrub belonging to the Lamiaceae family, also known as *Rabdosia rubescens* or “Suimiya” in Chinese, while its dried aerial parts are commonly referred to as “Donglingcao” in traditional Chinese medicine. The plant is characterized by erect, quadrangular stems and opposite, ovate leaves with crenate margins. The plant produces abundant secondary metabolites, mainly diterpenoids and phenolic acids. Among these, oridonin is the most representative diterpenoid active constituent of *I. rubescens* [1,2]. Oridonin is an *ent*-kaurane tetracyclic diterpenoid with a molecular formula of C_20_H_28_O_6_, first isolated from this plant in the 1960s. Its structure features an α,β-unsaturated ketone moiety, which is considered critical for its biological activity. It exhibits a broad range of pharmacological activities, including antibacterial [3,4], anti-inflammatory and antitumor activities [5]. In particular, oridonin has demonstrated anticancer activity against a variety of tumor types, such as lung, liver, breast, colorectal, and leukemia, through the modulation of multiple signaling pathways, including p53, MAPK, PI3K/Akt, and NF-κB, leading to cell cycle arrest and apoptosis induction in cancer cells [6]. Given the significant pharmacological value of oridonin, elucidating its tissue-level localization within the plant is of great importance for clarifying its biosynthetic pathways and guiding medicinal quality evaluation.

Epidermal-derived trichomes in plants fall into two major categories: non-glandular and glandular forms. Non-glandular trichomes provide a physical shield against herbivory and contribute to plant resilience to abiotic stressors including drought, chilling, and UV irradiation. In contrast, glandular trichomes act as chemical defense apparatuses and have been demonstrated to be the primary sites for the synthesis and storage of volatile terpenoids, including monoterpenes and sesquiterpenes [7]. Recently, non-volatile natural products stored in glandular trichomes have received growing research interest [8]. Non-volatile sugars and lipids increase leaf viscosity to block or kill insects, and certain non-volatile terpenoids have also been confirmed to accumulate in glandular trichomes [9]. In the Lamiaceae species *Salvia divinorum*, for instance, the neoclerodane diterpenoids (salvinorin A and related compounds) are secreted and accumulated as resin in the subcuticular space of peltate glandular trichomes [10]. In *Salvia miltiorrhiza*, diterpenoids are detectable exclusively in tissues bearing glandular trichomes, further supporting the close association between glandular trichomes and terpenoid secondary metabolites [11]. These lines of evidence suggest that glandular trichomes may serve as potential accumulation sites for oridonin in *I. rubescens*. Direct evidence from *I. rubescens* has further elucidated the morphological and functional characteristics of its glandular trichomes. Histochemical studies have classified the glandular trichomes into two types—peltate and capitate—with the former characterized by a short stalk and a large four-celled secretory head. Notably, peltate trichomes were proposed not only as sites of oridonin accumulation but also as sites of its biosynthesis [12].

Histochemical staining is a commonly used approach for the tissue localization of terpenoids. HCl–vanillin is a classic reagent for staining terpenoids and flavonoids, allowing differentiation among various types of secondary metabolites based on color reactions [13]. Staining of *I. rubescens* leaves revealed intense fluorescence signals in peltate glandular trichomes, whereas no appreciable fluorescence was observed in mesophyll cells [14,15]. It has thus been speculated that this fluorescence originates from secondary metabolites, particularly terpenoids, accumulated in the glandular trichomes, which may include not only volatile mono- and sesquiterpenes but also non-volatile diterpenoids. However, HCl–vanillin staining is not entirely specific to terpenoids, as flavonoids and other phenolic compounds may also yield positive reactions. Therefore, the fluorescence signals obtained by this method should be interpreted as preliminary evidence for terpenoid-enriched regions rather than definitive identification of specific terpenoid compounds.

With respect to extraction techniques, oridonin is soluble in organic solvents such as methanol, and ultrasonic extraction with methanol is a conventional method for oridonin extraction from dried *I. rubescens* leaves [16,17]. However, this method extracts the overall components of the leaf and is unable to distinguish the differential distribution of oridonin between glandular trichomes and mesophyll tissues. In contrast, rapid dichloromethane dipping enables targeted extraction of terpenoids stored beneath the cuticle of glandular trichomes; by briefly immersing leaves in dichloromethane, terpenoids can be specifically obtained from glandular trichomes without disrupting mesophyll cells [18,19]. This approach follows previously established protocols for the recovery of surface-localized trichome constituents in tobacco leaves [20] and has been similarly applied to the analysis of glandular trichome exudates in *Schizonepeta tenuifolia* [21]. After modification, it is also applicable to the extraction of diterpenoid components from glandular trichomes of *I. rubescens* [22].

In recent years, the development of single-cell and spatial omics technologies has enabled the in-depth investigation of plant metabolic heterogeneity at the molecular and spatial levels. High-resolution MSI provides a powerful tool for the in situ study of the spatial distribution of metabolites and lipids. The mainstream MSI techniques currently employed for spatial analysis of plant secondary metabolites include matrix-assisted laser desorption/ionization mass spectrometry imaging (MALDI–MSI) and DESI–MSI [23]. MALDI–MSI offers high spatial resolution but requires the application of an organic matrix to assist ionization, which may introduce matrix interference and increase sample preparation steps. DESI–MSI, in contrast, functions under ambient conditions and uses a charged solvent spray to desorb and ionize analytes directly from the sample surface, obviating matrix pretreatment. This approach offers rapid, sensitive, and accurate detection, and is especially suited for direct analysis of fresh plant tissues [24]. These techniques have been applied to the spatial distribution studies of various secondary metabolites in medicinal plants. MALDI–MSI has been used for the imaging of ginsenosides in *Panax ginseng* roots [25], phenolic acids and tanshinones in *S. miltiorrhiza* root, stem and leaf tissues [4,26], flavonoids and diterpenoids in sunflower linear glandular trichomes [27], and flavonoids in *Glycyrrhiza glabra* roots and stems [28], as well as *Ginkgo biloba* roots, stems and leaves [29]. DESI–MSI has been successfully applied to the spatial distribution of cannabinoids in glandular trichomes of *Cannabis sativa* leaves [30], diterpenoids in *S. miltiorrhiza* and related *Salvia species* [31], and artemisinin in *Artemisia annua* leaves [32]. In addition, LDI–FTICR–MSI has been used to localize sclareol accumulation in glandular trichomes of *Salvia sclarea* calyx epidermis [33], and contact printing combined with LDI–MS has enabled metabolic imaging of terpenoid acids and other metabolites in single glandular trichomes of wild tomato (*Solanum habrochaites*) leaves [34]. Collectively, these studies demonstrate that MSI has become a well-established platform for the in situ analysis of terpenoids and other secondary metabolites in plant glandular trichomes.

Despite the importance of oridonin as the principal bioactive diterpenoid in *I. rubescens*, its precise tissue localization within leaves remains unclear. In this study, DESI–MSI was employed to directly scan fresh, untreated *I. rubescens* leaves, in combination with histochemical staining and dichloromethane-targeted extraction coupled with HPLC quantitative analysis, to determine the cellular-level distribution of oridonin in leaves, thereby providing a theoretical basis for superior variety breeding and quality evaluation of *I. rubescens*. Furthermore, the antibacterial and cytotoxic activities of glandular trichome-targeted extracts, mesophyll extracts (following trichome removal), and whole-leaf extracts were evaluated. By comparing the activity differences among extracts from different sources, this study aimed to verify whether oridonin enriched in glandular trichomes constitutes the primary material basis for the antibacterial and cytotoxic activities of *I. rubescens*, thereby linking the tissue localization of oridonin to its pharmacological functions and providing functional evidence for the biological significance of glandular trichomes as storage sites for bioactive constituents in *I. rubescens*.

## 2. Materials and Methods

### 2.1. Plant Material

*I. rubescens* seeds were provided by the Jiyuan Research Institute of Zhengzhou University (Jiyuan, China). The species identity was confirmed by Prof. Jinyong Huang and Prof. Caipeng Yue at the Zhengzhou University. The plant material used in this study was obtained from hydroponic culture in a controlled growth chamber. The cuttings or seedlings were cultivated in Hoagland nutrient solution (Hopebio, Qingdao, China) under constant temperature conditions at 24 °C, with a photoperiod of 16 h light and 8 h dark.

### 2.2. Histochemical Staining

To prepare the HCl–vanillin staining reagent, 0.1 g of vanillin (Macklin, Shanghai, China) was dissolved in 100 mL of hydrochloric acid. Freshly harvested leaves were submerged in the staining solution for 2 min, subsequently mounted on glass slides, and air-dried before observation. Fluorescence was examined using a fluorescence microscope (Olympus DP53, Olympus Corporation, Tokyo, Japan), with images captured in both bright-field and dark-field modes. To examine the fluorescence distribution in internal tissues, leaves were torn to expose the mesophyll, or transversely sectioned, prior to staining and observation. HCl–vanillin staining was selected as an initial histochemical approach for the preliminary localization of terpenoids and other secondary metabolites in plant tissues [12,13,14]. However, this reaction is not specific to diterpenoids and serves as a preliminary detection of the distribution of constituents in *I. rubescens* leaves. All staining procedures were performed with at least three biological replicates, and multiple fields of view were examined per replicate to ensure representative observations. Representative images were presented.

### 2.3. DESI–MSI Analysis of Oridonin Distribution

Fresh *I. rubescens* leaves were cut to the right size to avoid the large veins, and were glued on the glass slide with double-sided adhesive tape. Both abaxial and adaxial surfaces were examined separately. Intact leaves were compared with those in which the protruding mesophyll tissue covering the glandular trichomes had been gently scraped away using a razor blade, leaving the underlying glandular trichomes exposed on the leaf surface. The adhesive tape served solely as a mounting medium to secure the leaf samples during mass spectrometry imaging and did not interfere with the analysis. The distribution of oridonin was scanned on a DESI–Tof mass spectrometer with a DESI ion source (Waters Corporation, Milford, MA, USA). High voltage was applied to generate charged solvent droplets, which were directed onto the leaf surface to facilitate analyte desorption and ionization. As an ambient ionization technique, DESI enables direct desorption and ionization of analytes from the sample surface under atmospheric pressure without the need for matrix pretreatment [35]. The signals were collected and transferred to the mass spectrometer to create mass spectrum images. Optical images of the leaf surface were captured prior to MSI analysis to guide the selection of regions of interest, and the ion images were subsequently overlaid with the optical images for spatial correlation. The parameters were: pixel size 100, spray solvent methanol (Merck, Darmstadt, Germany, HPLC grade), flow rate 2 μL/min, mass spectrometry scanning under positive ion mode, capillary voltage 4.5 kV, and *m*/*z* 50–600. MS images were reconstructed from raw data files, with leucine enkephalin (LE) serving as the lock mass for high-resolution calibration. Ion images for oridonin were generated at *m*/*z* 387.15. Compared with standard oridonin, the concentration was 800 mg/L. All staining and imaging experiments were performed with at least three biological replicates, and representative images are presented.

### 2.4. Extraction of Oridonin from Different Leaf Fractions

For oridonin extraction from different leaf parts, fresh *I. rubescens* leaves (72 g) were randomly divided into three biological replicates (24 g each). For each replicate, the 24 g fresh leaves were further split into two equal portions (12 g each), one portion was used for sequential extraction of glandular trichome compounds followed by mesophyll compounds from the same leaf material, and the other portion was used directly for whole-leaf extraction. All extracts were finally reconstituted in equal volumes of methanol prior to HPLC analysis, enabling direct comparison of oridonin content across the three fractions. A total of 12 g of the whole leaves were quickly dipped in a beaker containing 60 mL dichloromethane (Macklin, Shanghai, China, analytical grade) for 2 s, repeated 6 times, and then immersed in a second beaker of 40 mL dichloromethane for 2 s and repeated 3 times. The brief dipping time was chosen to restrict solvent action to the trichome surface layer, thereby enabling selective separation of trichome-derived compounds from mesophyll constituents. The two portions of dichloromethane were pooled and allowed to evaporate in a fume hood. The dried residue was re-dissolved in 30 mL methanol with ultrasonication for 10 min. Oridonin concentration in the glandular trichome extract was quantified by HPLC. The procedure was repeated three times as biological replicates. Following the removal of glandular trichome compounds by dichloromethane dipping, the same leaf portions were oven-dried at 45 °C to constant weight. After drying to constant weight, approximately 3 g, the leaves were thoroughly ground. A total of 30 mL of methanol was added to 1:10 for ultrasonic extraction for 10 min, and the concentration of oridonin was detected by HPLC, which was recorded as extracts in mesophyll. The above operation was repeated three times for biological repeat. For whole-leaf extraction, the remaining 12 g fresh leaf portion was dried at 45 °C to constant weight and ground to a fine powder. After drying to constant weight (approximately 3 g), the leaves were fully ground. The dried leaves were thoroughly ground and extracted with 30 mL methanol (1:10, *w*/*v*) by ultrasonication for 10 min. The concentration of oridonin was detected by HPLC, which was recorded as the extracts of complete leaves. The above operation was repeated three times for biological repeat.

### 2.5. HPLC Analysis of Oridonin

The HPLC conditions for oridonin quantification were established based on previously reported methods with minor modifications [36,37]. Prior to injection, all extract solutions were filtered through a 0.22 μm syringe filter and transferred to HPLC vials. Quantification of oridonin was performed on an Agilent–1100 HPLC system (Agilent Technologies, Santa Clara, CA, USA) fitted with an Inertsustain C18 column (5 μm, 4.6 mm × 150 mm, GL Sciences Inc., Tokyo, Japan). The column temperature was set at 30 °C. The mobile phase consisted of methanol and water (50:50, *v*/*v*). Injection volume: 5 μL. Flow rate: 1 mL/min. The detection wavelength was 238 nm. A series of solutions of oridonin were prepared and the standard curve was drawn. A calibration curve was established with peak area (y) plotted against oridonin concentration (x), yielding the equation y = 13.909x (R = 0.9996). The linear range of oridonin was 5–1200 mg/L.

### 2.6. Antibacterial Activity Assay

Antibacterial efficacy was assessed via the Kirby–Bauer disk diffusion assay, following the standard protocol recommended by the Clinical and Laboratory Standards Institute (CLSI). This standardized disk diffusion method is routinely applied in microbiological research and instructional settings, as it enables consistent evaluation of antimicrobial susceptibility through measurement of inhibition zone diameters [38]. Five indicator strains were used for the assay: *B. subtilis*, *M. luteus*, *S. aureus*, *Escherichia coli* and *Salmonella* spp. All bacterial strains were obtained from the laboratory stock of Zhengzhou University (Zhengzhou, China). Lyophilized bacterial powders were reconstituted in Luria–Bertani (LB) liquid medium and cultured at 37 °C with shaker. The bacterial liquid was drawn on LB solid medium and cultured in 37 °C incubator for 24 h. The single colony was selected and placed in LB liquid medium, cultured in 37 °C shaker for 6 h, and then diluted to prepare the bacterial suspension equivalent to 0.5 McIntosh unit. The circular filter paper was cut with a 6 mm diameter punch, sterilized in a high-temperature steam sterilization pot (121 °C, 30 min) and dried for use. The diluted bacterial suspension was coated on LB solid medium, waiting for 3 min for the surface liquid to evaporate. The sterile filter paper was pasted on the appropriate position on the surface of the medium. The extracts of glandular trichomes, extracts of mesophyll and extracts of complete leaves of 15 μL each were added to the center of the filter paper, and the standard solution of methanol, 800 mg/L oridonin and 5 mg/mL kanamycin were set. After 5 min, it was inverted and incubated at 37 °C for 18–24 h, and then the diameter of the bacteriostatic zone was determined. The diameters of the inhibition zones were measured to the nearest millimeter using a vernier caliper. The susceptibility levels were determined based on the interpretive criteria established in a prior report that utilized an identical experimental protocol [39], as summarized in Table 1. The three extracts and oridonin standard substance (Olchemim Ltd., Olomouc, Czech Republic, >99% purity) were respectively configured as 200 mg/L, and each 20 μL was taken to repeat the above inoculation operation.

### 2.7. Cytotoxicity Assay

The extracts were freeze-dried into powder and then 20 mg/mL dimethyl sulfoxide (DMSO, Solarbio, Beijing, China) solution was prepared at 4 °C. A549 human alveolar basal epithelial adenocarcinoma cells were seeded into 96-well plates at a density of 5000 cells per well. On the next day, 0.5 μL gradient concentration compound solution were added to 100 μL culture medium and incubated in the incubator for 36 h. It was washed twice with phosphate-buffered saline (PBS), and the medium containing Cell Counting Kit–8 (CCK–8, Sigma-Aldrich, St. Louis, MO, USA) was added to 96-well plate. After 1 h, the absorbance value of 450 nm was detected and the inhibition rate was calculated.

### 2.8. Statistical Analysis

Statistical analysis was performed using GraphPad Prism 5 (GraphPad Software, San Diego, CA, USA). All data were expressed as the mean ± standard deviation (SD) from at least three independent biological replicates. Statistical significance between multiple groups was determined by one-way analysis of variance (ANOVA) followed by Tukey’s post hoc test. A value of *p* < 0.05 was considered statistically significant.

## 3. Results

### 3.1. Fluorescence Imaging Analysis of I. rubescens Leaf

**Figure 1:**
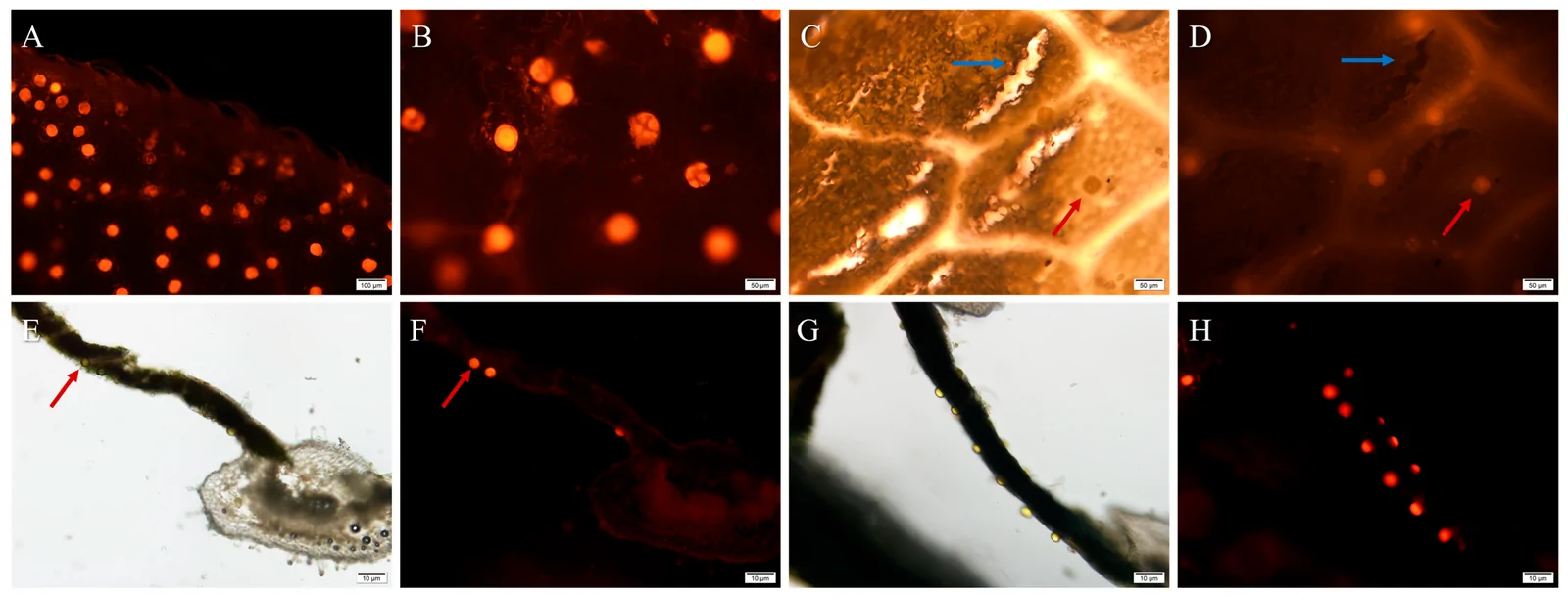
Histochemical localization of terpenoids in *I. rubescens* leaves using HCl–vanillin staining. Fluorescence distribution at the leaf margin showing strong orange–red fluorescence specifically in glandular trichomes, with no detectable signal in non-glandular trichomes (**A**). Enlarged view demonstrating that the fluorescence is restricted to the glandular trichome, whereas the surrounding mesophyll cells exhibit no positive reaction (**B**). Paired bright-field (**C**) and fluorescence (**D**) images of the same leaf tear site. The blue arrow indicates non-fluorescent mesophyll cells, and the red arrow indicates a fluorescent glandular trichome. Paired bright-field (**E**,**G**) and fluorescence (**F**,**H**) images of the same leaf transverse sections. The red arrow indicates glandular trichomes that emit specific fluorescence, while the mesophyll and vascular tissues remain non-fluorescent. Bar: (**A**) = 100 μm, (**B**–**D**) = 50 μm, (**E**–**H**) = 10 μm.

To visualize the spatial distribution of terpenoids, HCl–vanillin staining was performed on *I. rubescens* leaves. Strong specific fluorescence signals were exclusively detected in the peltate glandular trichomes, while no fluorescence was observed in the non-glandular trichomes, mesophyll cells, or vascular tissues (Figure 1). This was confirmed in both leaf surface examinations and transverse sections. Although HCl–vanillin staining is not specific to terpenoids and may also react with flavonoids and other phenolic compounds, the positive signals in glandular trichomes indicate the presence of terpenoid-like compounds in these structures, while the absence of detectable fluorescence in mesophyll tissues suggests that terpenoids, if present, are at substantially lower levels. Nevertheless, given the limited specificity of this histochemical method, these results serve as preliminary localization evidence and require further validation by more targeted analytical approaches.

### 3.2. Imaging Analysis of DESI Signal of I. rubescens Leaf

**Figure 2:**
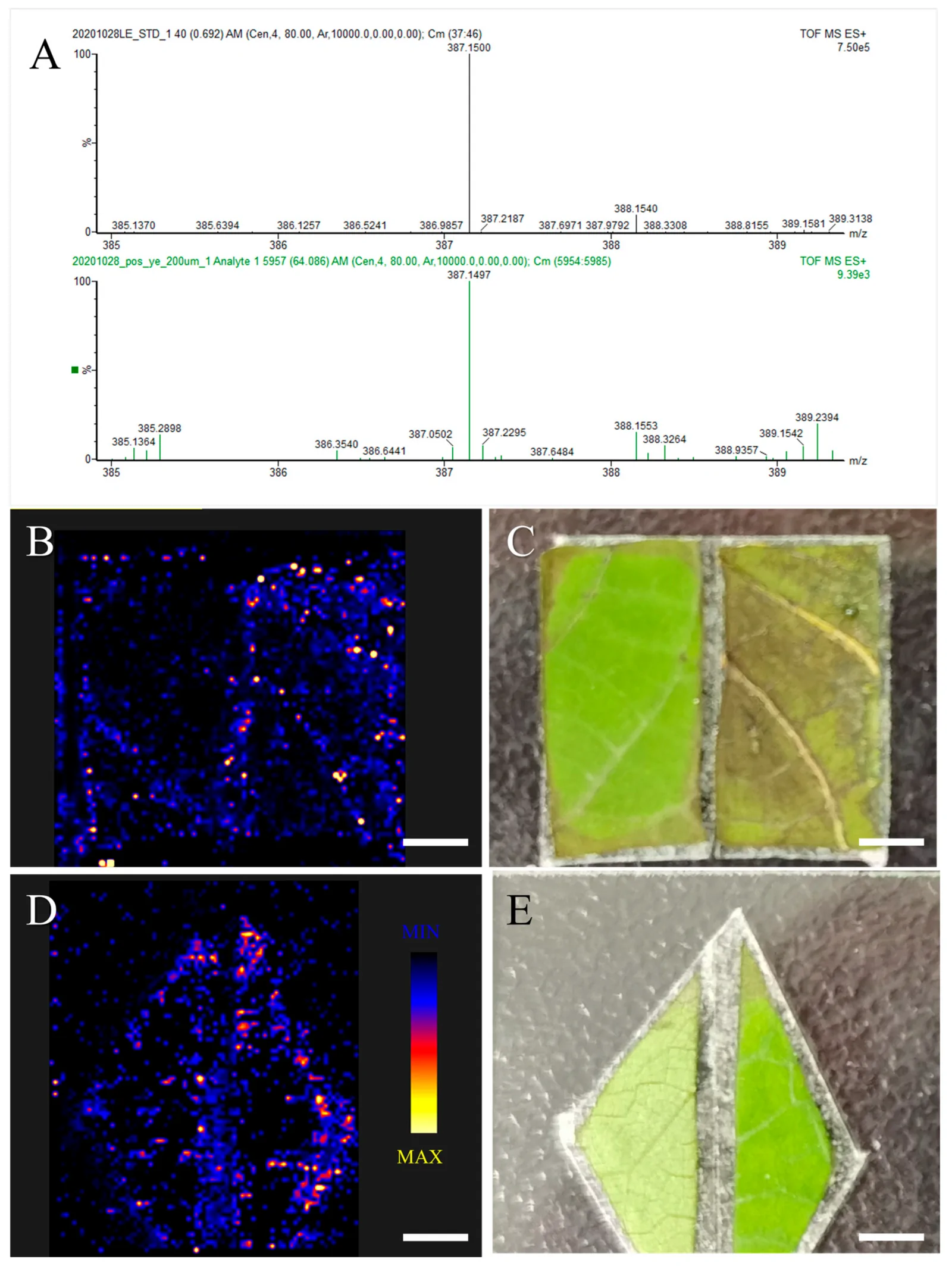
Spatial distribution of oridonin in *I. rubescens* leaves visualized by DESI–MSI. Representative mass spectra showing the characteristic ion peak of oridonin at *m*/*z* = 387.15 in both the standard (upper) and sample (lower) extracts (**A**). DESI–MSI ion image (**B**) and the corresponding optical image (**C**) of an *I. rubescens* leaf. The left half represents the intact leaf, while the right half was scraped to remove protruding mesophyll and expose the underlying glandular trichomes. The ion signals show a punctate pattern that closely matches the spatial distribution of the glandular trichomes. DESI–MSI ion image (**D**) and the corresponding optical image (**E**) of fresh leaves, displaying the abaxial (left) and adaxial (right) surfaces, respectively. Consistent punctate ion signals are present on glandular trichomes on both surfaces. Bar = 3 mm.

To further validate the tissue-specific localization of oridonin suggested by histochemical staining, DESI–MSI was employed to directly map the in situ distribution of oridonin on fresh *I. rubescens* leaves. The MSI results revealed that the characteristic ion signal of oridonin (*m*/*z* = 387.15) exhibited a punctate distribution pattern, which closely resembled the spatial arrangement of glandular trichomes on the leaf surface (Figure 2B–E). Because the glandular trichomes are embedded in the leaf epidermis, resulting in an uneven leaf surface, intact leaves were compared with those that had been scraped to remove protruding mesophyll and expose the underlying glandular trichomes. The results showed that after removing the mesophyll to flatten the leaf surface, the ion signals at the glandular trichome regions were notably enhanced, whereas almost no signal was detected in the directly exposed mesophyll cells. Furthermore, the density of glandular trichomes on the adaxial surface was higher than that on the abaxial surface, and the DESI–MSI images revealed a correspondingly denser distribution of punctate signals on the adaxial surface, showing high spatial consistency between the anatomical features and the mass spectrometric signals. Collectively, these DESI–MSI findings provide in situ mass spectrometric imaging evidence indicating that oridonin primarily originates from and is stored in the glandular trichomes of *I. rubescens* leaves.

### 3.3. HPLC Determination of Oridonin Extracted from Different Parts of Leaves

**Figure 3:**
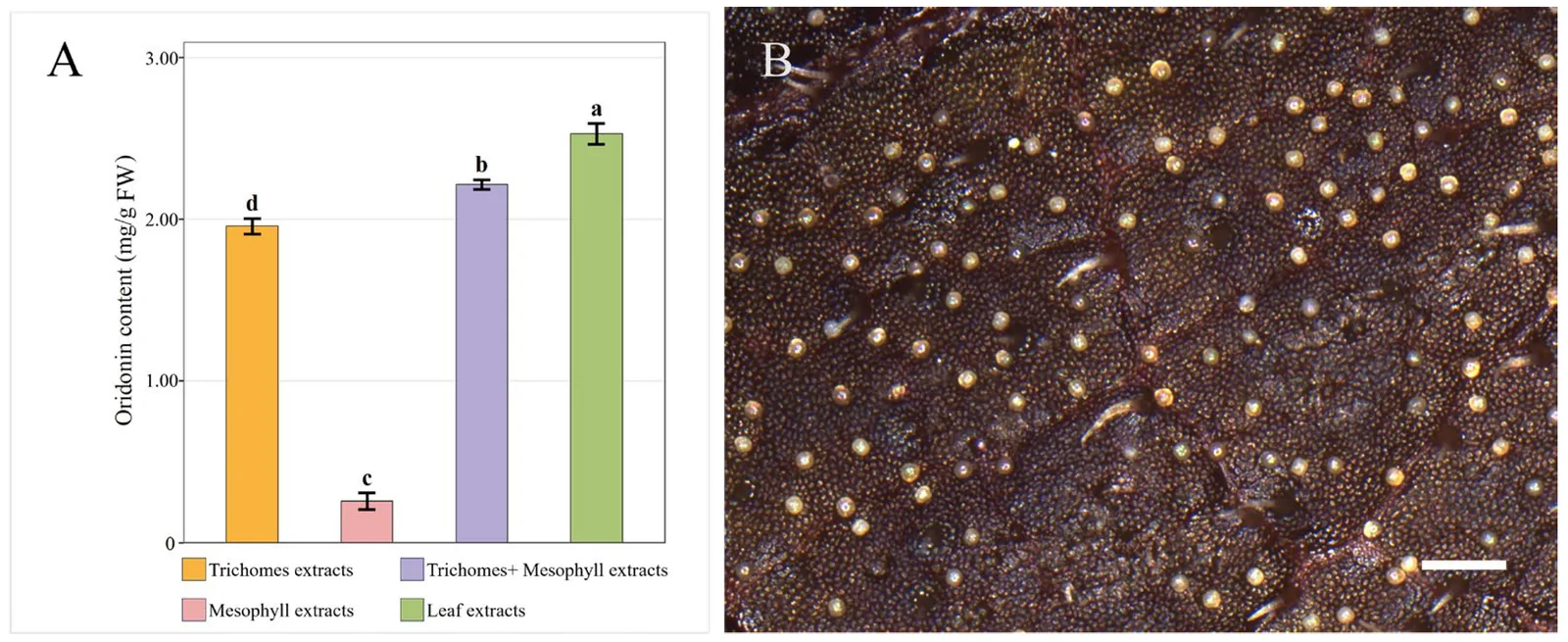
Extraction and quantitative analysis of oridonin from glandular trichomes of *I. rubescens* leaves. Comparison of oridonin concentration in different treatment (**A**). Data are presented as the mean ± SD (n = 3). Different lowercase letters above the bars indicate significant differences among the extraction treatments at *p* < 0.05. Morphology of *I. rubescens* leaves after rapid dipping in dichloromethane (**B**). The image clearly shows the punctate distribution of glandular trichomes on the leaf epidermis and demonstrates that the leaf surface morphology, including the integrity of mesophyll cells, remained intact after the brief dichloromethane treatment; bar = 100 μm.

To quantify the spatial distribution of oridonin, targeted dichloromethane dipping was combined with HPLC analysis (Figure 3). The rapid dichloromethane dipping method enabled selective extraction of oridonin from glandular trichomes. As shown in Figure 3B, the treated leaf surface retained its structural integrity, with glandular trichomes clearly visible as punctate protrusions on the epidermis, confirming that the brief solvent treatment did not cause apparent damage to the leaf tissues. The oridonin concentration in the glandular trichome extract was 1.958 ± 0.048 mg/g FW, accounting for 77.44% of that in the whole-leaf extract (2.528 ± 0.065 mg/g FW). In contrast, the mesophyll extract contained only 0.258 ± 0.052 mg/g FW (10.20%). The raw data of oridonin content in different leaf fractions are provided in Supplementary Table S1. The sum of oridonin content in the glandular trichome and mesophyll extracts accounted for 87.64% of the whole-leaf extract, suggesting an approximately 12% loss during the rapid dichloromethane dipping process, which may be attributed to trichome rupture or incomplete transfer.

Notably, while histochemical staining and DESI–MSI predominantly visualized highly concentrated signals in the glandular trichomes, the quantitative HPLC results further revealed the presence of trace amounts of oridonin in the mesophyll. This discrepancy suggests that the oridonin concentration in mesophyll cells is relatively low, falling below the detection thresholds of fluorescence microscopy and visual limits in MSI, whereas HPLC provides the necessary sensitivity for trace quantification. Nevertheless, the significantly higher content of oridonin in glandular trichomes compared with that in the mesophyll supports the conclusion that glandular trichomes serve as the primary storage site for oridonin in *I. rubescens* leaves.

### 3.4. Antibacterial Activity of the Extracts

**Figure 4:**
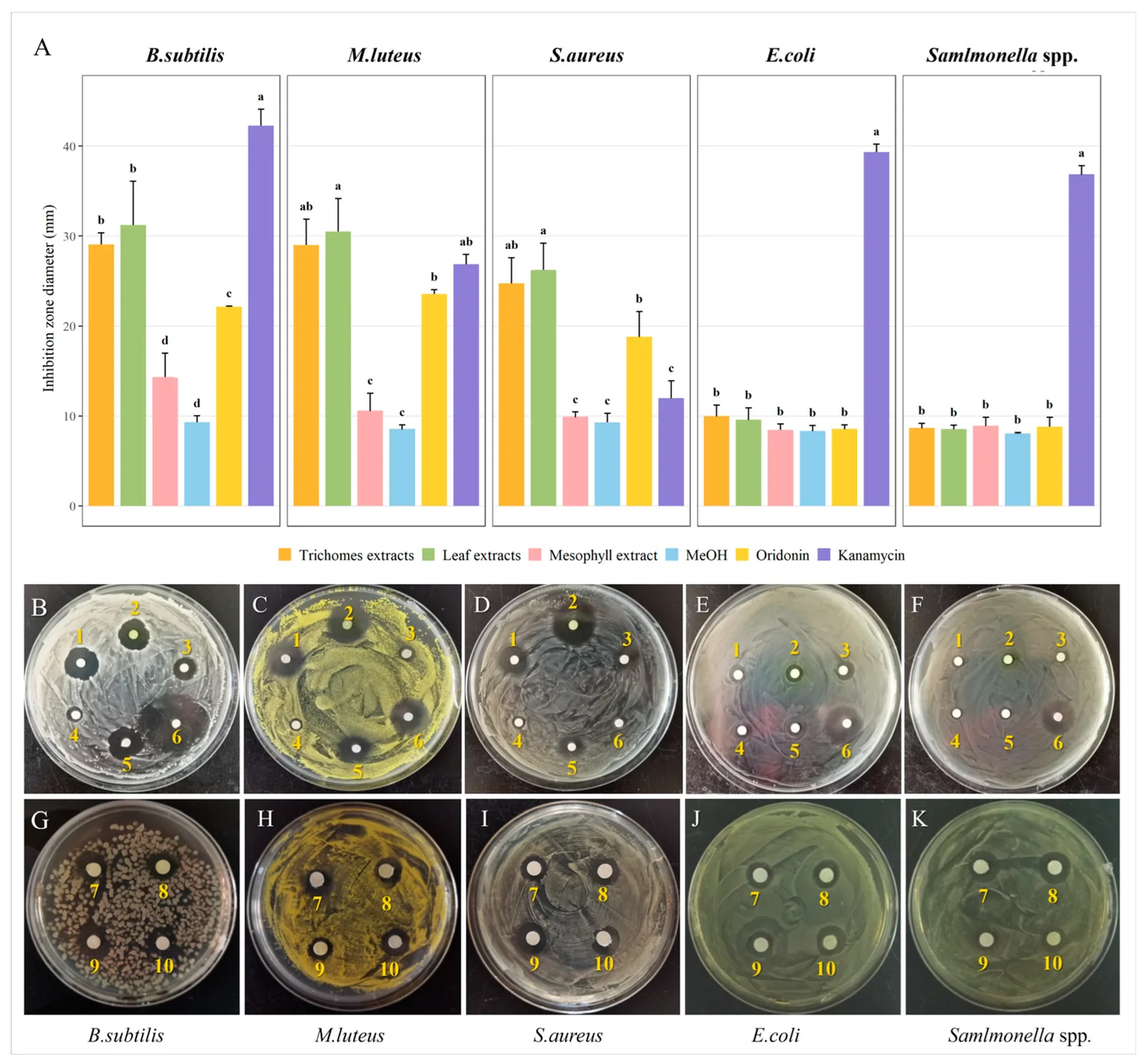
Bacteriostatic experiment of different extracts and corresponding reagents of *I. rubescens*. Comparison of inhibition zone diameter of five standard bacteria (**A**). Data are presented as the mean ± SD (n = 3). Different lowercase letters above the bars denote significant differences among different extract treatments within the same bacterial strain at *p* < 0.05. Treatments sharing the same letter are not significantly different. Letters are not comparable across different bacterial strains. Bacteriostatic experiment of different concentration extracts. *B. subtilis* (**B**), *M. luteus* (**C**), *S. aureus* (**D**), *E. coli* (**E**), *Salmonella* spp. (**F**); medicament on filter paper: 1. Dichloromethane extracts oridonin from leaf glandular trichomes. 2. Complete leaf extract. 3. Mesophyll extract. 4. Methanol. 5. Oridonin. 6. Kanamycin. Inhibition test of the extracts at the same concentration against different standard bacterial strains. (**G**) *B. subtilis*, (**H**) *M. luteus*, (**I**) *S. aureus*, (**J**) *E. coli*, (**K**) *Salmonella spp*. The filter paper: 7. Dichloromethane extracts oridonin from leaf glandular trichomes. 8. Complete leaf extract. 9. Oridonin. 10. Mesophyll extract.

The *I. rubescens* whole-leaf extract showed moderate sensitivity against *B. subtilis* and *M. luteus*, and slight sensitivity against *S. aureus*, but exhibited no inhibitory activity against the Gram-negative strains *E. coli* and *Salmonella* spp. (Table 2). Notably, the antibacterial profiles of the glandular trichome extracts were comparable to those of the whole-leaf extracts across all tested strains. In contrast, the mesophyll extracts displayed dramatically reduced activity, exerting almost no inhibitory effect on the three susceptible Gram-positive bacteria. This diminished activity was directly correlated with the significantly lower oridonin content in the mesophyll extracts (Figure 4). Additionally, no significant difference in inhibition zone diameters was found between the extracts and the oridonin standard at equivalent concentrations, suggesting that other co-extracted constituents did not affect the antibacterial efficacy. The raw data of inhibition zone diameters for all tested strains are provided in Supplementary Table S2. Collectively, these findings suggest that oridonin serves as the primary antibacterial component in *I. rubescens*.

### 3.5. Cytotoxicity of the Extracts

**Figure 5:**
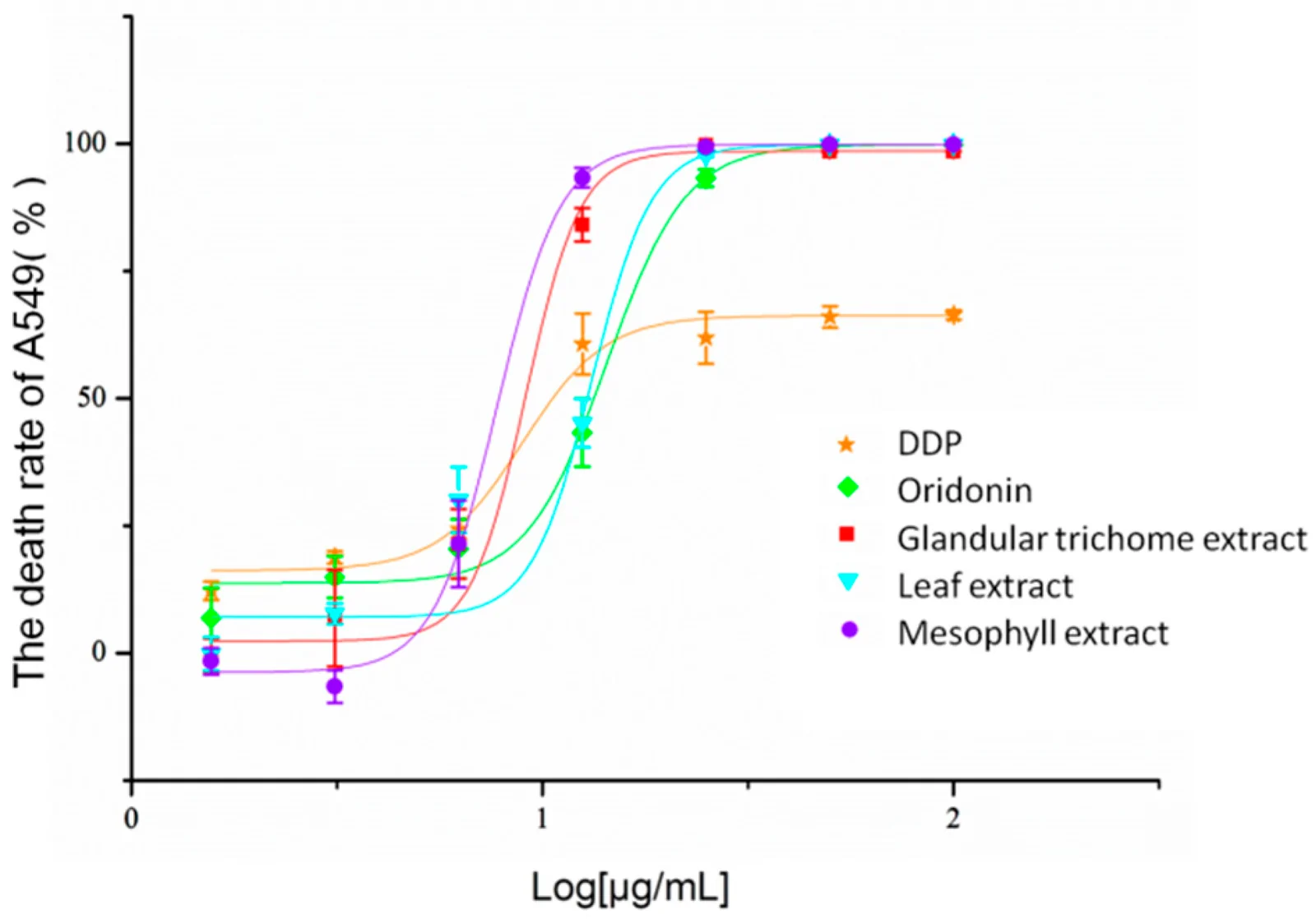
Cytotoxicity of extracts from different treatment and standard substance (DDP and oridonin) on A549.

The cytotoxic effects of the different extracts on A549 cells were evaluated using the CCK–8 assay (Figure 5). At lower concentrations, the cell death rates induced by the treatments ranked in the following descending order: the positive control cisplatin (DDP, Macklin, Shanghai, China), followed by oridonin, the whole-leaf extract, the glandular trichome extract, and lastly the mesophyll extract. However, at higher concentrations, all *I. rubescens* extracts induced nearly 100% cell death, whereas DDP reached a plateau at approximately 60% inhibition. The raw data of cell viability in the cytotoxicity assay are provided in Supplementary Table S3. As presented in Table 3, The half-maximal inhibitory concentrations (LD_50_) of the extracts and controls were determined. Based on the LD_50_ values, the overall cytotoxic potency of the tested samples ranked in descending order as follows, mesophyll extract, glandular trichome extract, DDP, whole-leaf extract, and oridonin, with lower LD_50_ values corresponding to higher cytotoxicity. This phenomenon suggests that the chemical composition of *I. rubescens* leaves is complex, and other cytotoxic components may exist, warranting further investigation to identify the active constituents. Oridonin exhibits cytotoxic activity against A549 cells, supporting its potential for further development as an anticancer agent.

## 4. Discussion

The results of histochemical staining, DESI–MSI in situ imaging, and targeted HPLC quantitative analysis are consistent with the view that, from histological, spatial distribution, and quantitative perspectives, oridonin exhibits a distinct compartmentalized distribution pattern in *I. rubescens* leaves, with peltate glandular trichomes serving as its primary accumulation site. Histochemical staining with HCl–vanillin provided initial evidence that terpenoid-like compounds are enriched in peltate glandular trichomes, as intense orange–red fluorescence signals were detected exclusively in the peltate glandular trichomes, whereas no specific fluorescence response was observed in mesophyll cells or vascular tissues. Given that HCl–vanillin staining is not entirely specific to terpenoids, these results should be interpreted as preliminary localization evidence requiring further confirmation. DESI–MSI analysis further revealed that the characteristic ion peak of oridonin (*m*/*z* 387.15) displayed a punctate distribution pattern on the leaf surface, which closely matched the arrangement of glandular trichomes. Quantitative HPLC analysis revealed that the oridonin content in the glandular trichome extract accounted for 77.44% of that in the whole-leaf extract, while the mesophyll extract contained only 10.20%. Collectively, these three lines of evidence, histochemical localization, spatial imaging, and quantitative analysis, are consistent with the conclusion that oridonin exhibits a distinct compartmentalized distribution pattern in *I. rubescens* leaves, with peltate glandular trichomes serving as its primary accumulation site.

This distribution pattern has also been reported in other Lamiaceae species. In *Salvia divinorum*, neoclerodane diterpenoids (salvinorin A) have been shown to be stored as resin in the subcuticular space of peltate glandular trichomes [9], and in *Salvia miltiorrhiza*, diterpenoids have been detected exclusively in tissues bearing glandular trichomes [10]. The findings of the present study further extend this general pattern to *I. rubescens*, providing additional evidence for the hypothesis that glandular trichomes serve as conserved storage sites for non-volatile diterpenoids within the Lamiaceae family.

Notably, trace amounts of oridonin were detected in the mesophyll extract by HPLC (10.20%), whereas no significant signals were observed in mesophyll tissues by histochemical staining or DESI–MSI. This discrepancy may be attributed to the differences in sensitivity among the detection methods. HPLC, as a highly sensitive quantitative technique, is capable of detecting trace components, whereas the visualization thresholds of histochemical staining and mass spectrometry imaging are relatively higher. Alternatively, the trace amounts of oridonin detected in the mesophyll may not originate from de novo synthesis or storage within the mesophyll itself, but rather from diffusion during sample preparation or cross-contamination during extraction. Regardless of the explanation, the 77.44% contribution from glandular trichomes, together with the negative results obtained from the two in situ visualization methods, supports the conclusion that glandular trichomes constitute the primary storage site for oridonin in *I. rubescens* leaves.

The principle of the rapid dichloromethane dipping method is based on the rapid dissolution of terpenoids stored beneath the glandular trichome cuticle by dichloromethane, while the brief immersion period does not disrupt the integrity of mesophyll cells or the epidermal structure [18,19,20,21]. Following dichloromethane treatment, the leaf surface structure remained intact, with glandular trichomes still clearly visible on the epidermis (Figure 3B), indicating the tissue specificity of this extraction method. The oridonin content in the glandular trichome extract accounted for 77.44% of that in the whole-leaf extract, whereas the mesophyll extract contained only 10.20%, with a statistically significant difference between the two. The sum of oridonin content in the glandular trichome and mesophyll extracts accounted for 87.64% of that in the whole-leaf extract, suggesting that approximately 12% of oridonin was lost during the dichloromethane dipping process, which may be attributed to trichome rupture or incomplete transfer of oridonin from the subcuticular space. It should be acknowledged that the extraction procedures for glandular trichome compounds and mesophyll compounds differed in solvent and duration, which may have influenced the absolute recovery of oridonin from each fraction. The dichloromethane dipping method was specifically designed to target cuticle-stored trichome constituents, while methanol ultrasonication was applied to extract the remaining oridonin in mesophyll tissues after trichome removal. Although the two procedures are not directly comparable for exhaustive extraction, the chosen conditions were based on established methods targeting different leaf compartments [20,21]. Consequently, the oridonin content reported for each fraction should be interpreted as a relative estimate of its distribution rather than an absolute quantification of total oridonin in the respective tissue. Nevertheless, this method successfully achieved effective separation of trichome-derived oridonin from mesophyll components, providing a simple and reliable tool for targeted studies of glandular trichome metabolites in *I. rubescens*. And the consistency between the HPLC quantitative data and the histochemical and DESI–MSI localization results strengthens the qualitative conclusion that glandular trichomes are the primary storage site of oridonin in *I. rubescens* leaves, despite the methodological differences in extraction.

The antibacterial activity evaluation revealed that both the glandular trichome extract and the whole-leaf extract exhibited significant inhibitory effects against *B. subtilis*, *M. luteus*, and *S. aureus*, with largely consistent antibacterial spectra, whereas the mesophyll extract showed almost no inhibitory activity against the above three Gram-positive bacteria. The inhibition zone diameters were positively correlated with the oridonin content in the extracts, the glandular trichome extract, which contained the highest oridonin content, also exhibited the strongest antibacterial activity, while the mesophyll extract, which contained the lowest oridonin content, showed the weakest activity. This correlation suggests that oridonin stored in glandular trichomes may serves as a major contributor to the antibacterial activity of *I. rubescens*. However, as formal correlation analysis and additional assays were not performed, this interpretation should be considered preliminary. Further studies, including dose–response comparisons and isogenic strain panels, would be required to confirm the direct causal role of oridonin in the observed antibacterial effects. None of the three extracts exhibited obvious inhibitory effects against *Escherichia coli* or *Salmonella* spp., suggesting that the antibacterial spectrum of oridonin is selective for Gram-positive bacteria. Notably, the kanamycin-resistant mutant of *S. aureus* remained sensitive to oridonin, implying that the antibacterial mechanism of oridonin differs from that of kanamycin, a property that warrants further investigation.

The cytotoxicity assay revealed more complex activity profiles. At higher concentrations, all *I. rubescens* extracts induced nearly 100% cell death in A549 cells, whereas cisplatin reached a plateau at approximately 60% inhibition, indicating that the cytotoxic potential of *I. rubescens* extracts at higher concentrations exceeded that of cisplatin. The LD_50_ values were ranked as follows: mesophyll extract (7.58 μg/mL) < glandular trichome extract (9.12 μg/mL) < cisplatin (10.47 μg/mL) < whole-leaf extract (12.30 μg/mL) < oridonin standard (12.88 μg/mL). This ranking presents an apparently paradoxical observation—the mesophyll extract, which contained the lowest oridonin content, exhibited the lowest LD_50_ value (i.e., the strongest overall cytotoxicity). One possible explanation for this phenomenon is that the mesophyll extract may contain other non-oridonin cytotoxic components, which might exert additive or synergistic effects at higher concentrations, thereby enhancing the overall cytotoxicity of the mesophyll extract. Alternatively, a concentration-dependent biphasic effect may exist in the mesophyll extract, strong cytotoxicity at higher concentrations and a mild cell-promoting effect at lower concentrations. The LD_50_ of the whole-leaf extract (12.30 μg/mL) was close to that of the oridonin standard (12.88 μg/mL), suggesting that the cytotoxicity of the whole-leaf extract is primarily contributed by oridonin, with relatively minor interference from other constituents. The chemical complexity of *I. rubescens* leaves is thus evident, and further work is required to isolate and identify the non-oridonin cytotoxic components in the mesophyll extract and to elucidate the mechanisms underlying the biphasic effect.

Taken together, the histochemical localization, mass spectrometry imaging analysis, and targeted extraction combined with HPLC quantification collectively suggest that oridonin is predominantly stored in the glandular trichomes of *I. rubescens* leaves. The oridonin stored in glandular trichomes constitutes the primary material basis for the antibacterial activity of *I. rubescens* and also serves as an important contributor to its cytotoxicity. This study provides a cellular-level theoretical basis for the quality evaluation of *I. rubescens*—using glandular trichome density or oridonin content as indicators—and offers a targeted direction for the breeding of high-oridonin-content varieties.

Although the present study provides evidence that glandular trichomes are the primary storage site for oridonin, whether they also possess the capacity for oridonin biosynthesis remains unclear. Recent transcriptomic studies have identified multiple differentially expressed genes potentially involved in oridonin biosynthesis in *I. rubescens*, and MSI technology has been explored for gene–metabolite spatial correlation analysis [40,41,42,43]. These tools offer promising avenues for future elucidation of the oridonin biosynthetic and regulatory networks within glandular trichomes. In addition, the phloem–xylem translocation mechanism of oridonin in planta warrants further investigation to achieve a comprehensive understanding of its source–sink relationship [44]. The integration of glandular trichome morphological characteristics, molecular regulatory elements, and metabolite transport pathways may provide more direct targets for the molecular breeding of *I. rubescens* varieties with high oridonin content.

## 5. Conclusions

In the present study, histochemical staining, DESI–MSI, and dichloromethane-targeted extraction coupled with HPLC quantitative analysis were employed to systematically determine, for the first time, the precise localization of oridonin in *I. rubescens* leaves. Fluorescence signals in response to HCl–vanillin staining were observed exclusively in glandular trichomes, with no detectable fluorescence in mesophyll cells. DESI–MSI analysis further confirmed that the characteristic ion signal of oridonin exhibited a punctate distribution pattern corresponding to the positions of glandular trichomes. Quantitative HPLC analysis revealed that oridonin content in the glandular trichome extract accounted for 77.44% of that in the whole-leaf extract, whereas the mesophyll extract contained only 10.20%, with statistically significant differences among the three extracts. Collectively, these convergent lines of evidence strongly suggest that oridonin is predominantly distributed in leaf glandular trichomes, which serve as its primary storage site in *I. rubescens*.

Functional activity evaluation revealed that both the glandular trichome extract and the whole-leaf extract exhibited significant antibacterial activity against *B. subtilis*, *M. luteus*, and *S. aureus*, and the diameter of the inhibition zone was positively correlated with oridonin content in the extracts, indicating that oridonin enriched in glandular trichomes constitutes the primary material basis for the antibacterial activity of *I. rubescens*. The cytotoxicity assay further demonstrated that high-concentration *I. rubescens* extracts exhibited marked cytotoxic effects on A549 cells, with half-maximal inhibitory concentrations comparable to or lower than that of cisplatin, further confirming the pharmacological activity of oridonin stored in glandular trichomes.

## Figures and Tables

**Table 1:** Drug sensitivity criteria of K–B method.

The Judgment of Drug Sensitivity	Inhibition Zone Diameter, IZD (mm)
Highly sensitive	≥32
Moderate sensitivity	≥22, <32
Mild sensitivity	≥20, <22
No inhibition	<20

**Table 2:** Antibacterial activities of different *I. rubescens* leaf extracts against five bacterial strains.

Species of Strains	Mean Inhibition Zone Diameter (mm)					
	Glandulr Trichome Extract	Leaf Extract	Mesophyll Extract	MeOH	Oridonin	Kanamycin
*B. subtilis*	29.08 ± 1.28 b	31.22 ± 4.88 b	14.23 ± 2.76 d	9.33 ± 0.68 d	22.13 ± 0.10 c	42.28 ± 1.81 a
*M. luteus*	29.00 ± 2.88 ab	30.47 ± 3.69 a	10.60 ± 1.94 c	8.57 ± 0.45 c	23.58 ± 0.46 b	26.88 ± 1.06 ab
*S. aureus*	24.73 ± 2.85 ab	26.20 ± 3.01 a	9.92 ± 0.53 c	9.28 ± 1.03 c	18.82 ± 2.79 b	11.97 ± 1.93 c
*E. coli*	9.98 ± 1.23 b	9.60 ± 1.31 b	8.43 ± 0.67 b	8.33 ± 0.61 b	8.57 ± 0.45 b	39.33 ± 0.87 a
*Salmonella* spp.	8.68 ± 0.51 b	8.55 ± 0.44 b	8.92 ± 0.95 b	8.08 ± 0.08 b	8.82 ± 1.03 b	36.85 ± 0.95 a

Note: Data are presented as the mean ± SD (n = 3). Different lowercase letters above the bars denote significant differences among different extract treatments within the same bacterial strain at *p* < 0.05. Treatments sharing the same letter are not significantly different. Letters are not comparable across different bacterial strains.

**Table 3:** LD_50_ of extracts from different methods and DDP to A549.

The Method of Treatments	LD_50_ (μg/mL)
Glandular trichome extract	9.12 ± 1.72
Mesophyll extract	7.58 ± 1.52
Leaf extract	12.30 ± 2.06
Oridonin	12.88 ± 1.99
DDP	10.47 ± 1.57

## Data Availability

The original contributions presented in this study are included in the article/Supplementary Materials. Further inquiries can be directed to the corresponding authors.

## Supplementary Materials

The following supporting information can be downloaded at https://www.mdpi.com/article/10.3390/cimb48090887/s1.
